# Advanced Extrauterine Pregnancy at 33 Weeks with a Healthy Newborn

**DOI:** 10.1155/2014/102479

**Published:** 2014-12-03

**Authors:** Tajudeen Dabiri, Guillermo A. Marroquin, Boleslaw Bendek, Enyonam Agamasu, Magdy Mikhail

**Affiliations:** Department of Obstetrics and Gynecology, Bronx Lebanon Hospital, Bronx, NY, USA

## Abstract

Abdominal pregnancy is a very rare form of ectopic pregnancy, associated with high morbidity and mortality for both fetus and mother. It is, and often, seen in poor resource nations, where early diagnosis is often a major challenge due to poor prenatal care and lack of medical resources. An advanced abdominal pregnancy with a good fetal and maternal outcome is therefore a more extraordinary occurrence in the modern developed world. We present a case of an abdominal pregnancy at 33.4 weeks in an individual with no documented prenatal care, who arrived in a hospital in the Bronx, in June 25th 2014, with symptoms of generalized, severe lower abdominal pain. Upon examination it was found that due to category III fetal tracing an emergent cesarean section was performed. At the time of laparotomy the fetus was located in the pelvis covered by the uterine serosa, with distortion of the entire right adnexa and invasion to the right parametrium. The placenta invaded the pouch of Douglas and the lower part of the sigmoid colon. A massive hemorrhage followed, followed by a supracervical hysterectomy. A viable infant was delivered and mother discharged on postoperative day 4.

## 1. Introduction

Symptoms of an abdominal pregnancy are very nonspecific and often include abdominal pain, nausea, vomiting, palpable fetal parts, fetal mal presentation, pain on fetal movement, and displacement of the cervix.

With remarkable advances in radiographic technology an early discovery of an extrauterine pregnancy should be a practicable endeavor. This is particularly important in a community where there are an increased number of immigrants from low resource nations [1].

The prevalence of ectopic pregnancy is 1-2% with 95% occurring in the fallopian tube. The incidence of abdominal pregnancy ranges from 1 : 1000 to 1 : 30,000 depending on the community but is most commonly seen in developing nations of the world [2, 3], which represent approximately 1–1.4% of all ectopic pregnancies alone [4–6]. The first documented case of abdominal pregnancy was reported in the year 1708, followed by numerous case reports particularly from middle and low income regions of the world [7]. Frequently, the diagnosis was made based on complications such as hemorrhage and abdominal pain at the time of laparotomy. Most often, the pregnancy did not survive and often resulted in extraction of the dead fetus with increased maternal mortality.

In the developed world, abdominal pregnancy is extremely rare and very few of such cases have been published in the last 10 years. It is unclear if abdominal pregnancy is a result of secondary implantation from an aborted tubal pregnancy or result of primary implantation from intra-abdominal fertilization. Associated risks for developing abdominal pregnancy are endometriosis, pelvic inflammatory disease, assisted reproductive techniques, tubal occlusion, and multiparity [8–10].

In view of rarity and lack of management guidelines of advanced abdominal pregnancy, we expose this case of abdominal pregnancy in order to present the symptoms associated that could lead to an early recognition and the successful management that resulted in a good maternal and fetal outcome.

## 2. Case Report

A 27-year-old G2P0010 at 33 weeks and 4 days by last menstrual period was brought in by Emergency System to the hospital on June 25th 2014, with complaints of severe abdominal pain of 1 hour duration. Patient was without medical or surgical history and had a termination of pregnancy before. Abdominal pain was generalized, 10 out of 10 in severity, and associated with vomiting. She denied any diarrhea, vaginal bleeding, or leakage of amniotic fluid. She had recently migrated from the Dominican Republic in May 2014 with no record of prenatal care.

On examination, patient was in visible pain with elevated blood pressure, maternal tachycardia, and bilious emesis. An abdominal examination revealed generalized tenderness with guarding and rebound and a fundal height of 34 cm. The fetal heart rate was category III with absent variability and repetitive late decelerations. A vaginal examination revealed a bulging pouch of Douglas with the presenting part deep in the pelvis: a short, firm, and closed cervix displaced anteriorly behind the pubic symphysis.

On the way to the operating room limited bed side sonogram revealed fetus in cephalic and a questionable placental location. A tentative diagnosis of uterine rupture versus concealed placental abruption was made proceeding with immediate abdominal delivery.

At the time of laparotomy, meconium stained amniotic fluid was seen upon entry to the peritoneal cavity. A fetus was located outside of the endometrial cavity covered only by the uterine serosa on the right side with a placenta attachment to the serosa of the uterus. The left ovary was unremarkable in appearance and an anatomical distortion of the right adnexa was appreciated. A large opening was noted on the posterior aspect of the serosa where the amniotic fluid was leaking.

**Figure 1:**
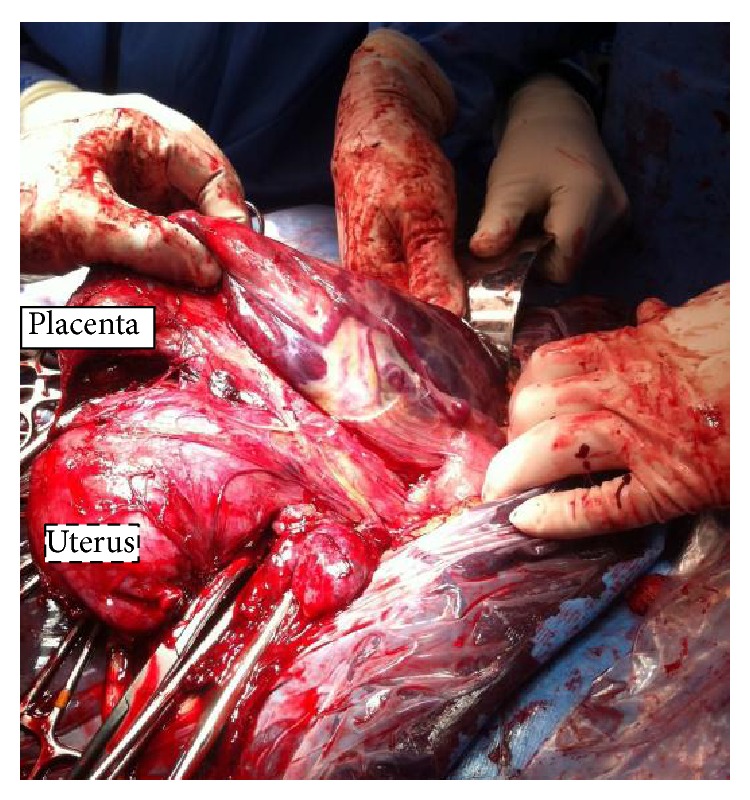
Representing placenta location and uterus after delivery of the baby, to note the size and the integrity of the uterus with a large placenta in the abdominal cavity.

An incision was made on the protruding serosa and a viable female infant was delivered via cephalic presentation with Apgar score of 9/9 at 1 and 5 minutes with weight of 2362 g. The uterus and placenta were exteriorized after delivery due to massive bleeding and distortion of the anatomy (Figure 1). On further inspection of the placenta, it was noted to invade the pouch of Douglas and lower part of the sigmoid colon and the right uterine serosa.

A massive hemorrhage protocol was initiated and an emergency back-up team was called. A general surgical consult was requested due to involvement of bowel. The decision was made to proceed on hysterectomy and removal of the placenta tissue due to continuous bleeding. The patient underwent supracervical hysterectomy and excision of the placenta tissue occupying the right side of the pelvic floor. Adhesiolysis from the sigmoid colon was performed by surgery with minimal damage to the serosa.

Intraoperatively, the patient received 6 units of packed red blood cells, 4 units of fresh frozen plasma, and one unit of platelets. Estimated blood loss was 3000 mL. The patient was then transferred to the ICU for further observation and extubated the following morning.

She was discharged home with the baby on day 4 after surgery. There was no evidence of anomaly documented in the baby. Mother and baby are doing well and currently being followed up closely.

A pathology report revealed that placenta with a segment of trivessel umbilical cord marked old infarct at fetal and maternal surfaces. Attached to the maternal surfaces are fibrous tissues with smooth muscle and dilated vessels. Focal endovasculopathy with luminal occlusion, focal amnion with squamous metaplasia with an attached stretched ovary and fragment of mostly chorionic villi.

The uterus was described as intact and weighed 300 g measuring 9.5 cm in length, 11 cm from cornua to cornua and 6 cm anterior posterior diameter with thick endometrial, decidual changes and focal autolysis, no chorionic villi or trophoblast are seen in the endometrium.

## 3. Discussion

Primary abdominal pregnancy refers to an extrauterine pregnancy where implantation of fertilized ovum occurs directly in the abdominal cavity while the secondary abdominal pregnancy is a tubal pregnancy that ruptures with reimplantation within the abdominal cavity usually resulting in tubal or ovarian damage [10].

In this report, the findings of recurrent pain throughout pregnancy especially during fetal movement, signs of peritonitis on day of presentation with free fluid in the abdomen, and findings of intraoperative distortion of the right ovary and fallopian tube are more indicative of a ruptured tubal pregnancy with a secondary implantation on the serosa and the right broad ligament. Nunyaluendo and Einterz [11], in a recent review of 163 cases of abdominal pregnancy, revealed that identification of this condition is often missed with only 45% cases diagnosed during the prenatal period. In this case, patient did not have any prenatal care and had history of intermittent pain throughout the pregnancy. Another factor to consider is the fact that she had a previous termination of pregnancy in the first trimester via suction curettage previously to this pregnancy in 2012 that could cause a defect in the uterus.

Interestingly, the most common symptoms in abdominal pregnancy are abdominal pain 100%, nausea and vomiting 70%, and general malaise 40% [12]. Our patient had sudden severe abdominal pain with vomiting one hour prior to presentation to the hospital. A high index of suspicion for possible rupture of uterus versus abdominal pregnancy should be always considered when the fetal parts are easily palpated on abdominal examination and signs and symptoms of an acute abdomen. However a vaginal examination revealed fetal head bulging through the pouch of Douglas displacing the cervix into the retropubic space as described before is a concerning finding.

An abdominal pregnancy is often associated with fetal deformities [13], such as facial and cranial asymmetry, joint abnormalities and limb deformity, and central nervous deformities in about 21% of cases. In our case, there was no evidence of deformity or abnormalities as per the team of pediatricians.

Bleeding from placental implantation site could be massive and life threatening and is often the most common cause of maternal mortality which can reach as high as 20–30%. The decision to remove or leave the placenta should depend on extent of the placentation particularly with the bowel and omental involvement as well as on the expertise of the surgeon. Because of increased postoperative morbidity and mortality, it is not advisable to leave the placenta in situ [13]. In this case, because of the involvement of the broad ligament on the right side with distortion of the ovary and tube on the same side and extension of part of the placenta to small portion of the sigmoid colon posteriorly the decision was made intraoperatively for a supracervical hysterectomy to obtain adequate hemostasis. In our case massive transfusion protocol was applied as per hospital protocol [14].

## 4. Conclusion

A high index of suspicion and recognition of signs and symptoms are therefore detrimental to diagnosis and guide to a prompt surgical emergency. In patients with acute symptoms and lack of prenatal care, abdominal pregnancy should always be a differential.

Prompt delivery of the fetus, followed by and control of hemorrhage and decision of placenta removal are the greatest challenges. Adequate personnel including anesthesia, pediatricians, and general surgeons may be necessary for a successful management.
